# Supplementary material for: Identification of photoperiod-induced specific miRNAs in the adrenal glands of Sunite sheep (Ovis aries)
Source: Front Vet Sci. 2022 Jul 22;9:888207. doi: 10.3389/fvets.2022.888207 (PMC9354845; doi:10.3389/fvets.2022.888207)
Supplement: Supplementary file 1 [file Data_Sheet_1.ZIP › Supplementary file/miRDeep2 file.zip!/miRDeep2 file/Novel_Structure/LP42A3/Novel_107.pdf]

Provisional ID	:	NC_019474.2_17841
Score total	:	2
Score for star read(s)	:	-1.3
Score for read counts	:	0
Score for mfe	:	1.7
Score for randfold	:	1.6
Score for cons. seed	:	
Total read count	:	144
Mature read count	:	144
Loop read count	:	0
Star read count	:	0

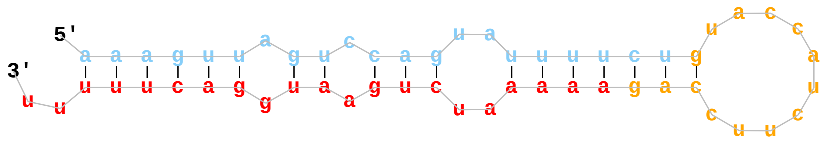

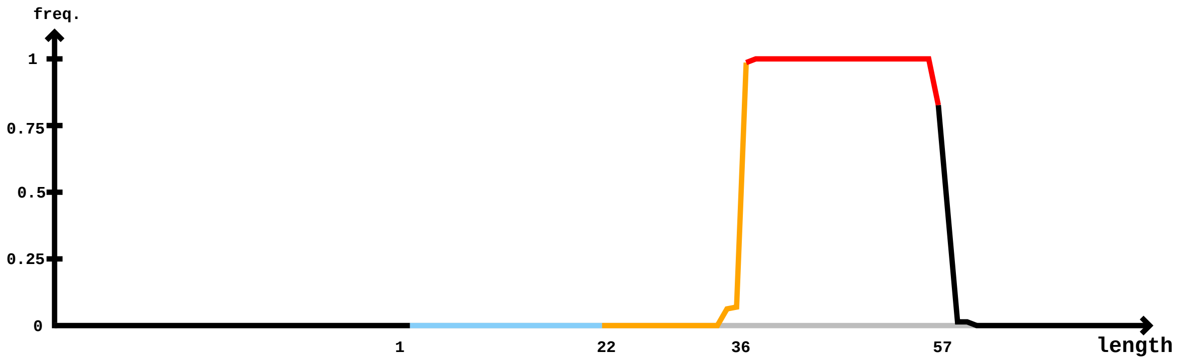

Star Mature

```
5'- ccugugauacuugugugaaauauuggauuggccaaaaaguuaguccaguauuuucuguaccaucuuccagaaaaaucugaauggacuuuuuggccaauccauauauauuugu  -3'   exp
    ................(((((((((((((((((((((((((.((.(((..(((((((.........)))))))..))).)).)))))))))))))))))))...))))))..   reads   mm   sample
    ....................................................................agaaaaaucugaaugAacuuuu......................   2       1    seq
    ....................................................................agaaaaaucugaaugAacuuuuu.....................   7       1    seq
    .....................................................................gaaaaaucugaaugAacuuuuu.....................   1       1    seq
    ......................................................................aaaaaucugaaugAacuuuu......................   23      1    seq
    ......................................................................aaaaaCcugaauggacuuuuu.....................   1       1    seq
    ......................................................................aaaaaucugaaugAacuuuuu.....................   65      1    seq
    ......................................................................aaaaaucugaauggacuuuuug....................   1       0    seq
    ......................................................................aaaaaucugaaugAacuuuuug....................   42      1    seq
    .......................................................................aaaaucugaaugAacuuuuuggc..................   2       1    seq
```
